# Some Impressions of Continental Hospitals

**Published:** 1914-02-07

**Authors:** 


### SOME IMPRESSIONS OF CONTINENTAL HOSPITALS.

By A TRAVELLING RESIDENT MEDICAL OFFICER.

Hospitals on the Continent, as in our country, present great contrasts according to their age and the enterprise of the controlling powers. To spend a little time in visiting representative institutions is a very profitable and instructive way of employing part of one's holiday leisure. But it would be unfair to compare the magnificent Rudolf Virchow Hospital in Berlin with the old hospital in the centre of Holland's capital. The Binnen Gasthuis in Amsterdam is, however, an instructive place to visit. Situated in a crowded part of the town and surrounded on all sides by buildings, one sees at once how the same difficulties of want of room for expansion and high cost of land are met with as in London. The pavilion system is out of the question here, and the high buildings with small open areas remind one forcibly of many of our Metropolitan hospitals. In the wards, however, things are rather different, and the visitor perceives that satisfactory, if pharisaical, feeling that here at least these things are done better at home.

Leaving aside the question of old floors and the dark corridors, the most striking feature is the small space between the beds. Judged by our own standards there exists a considerable degree of overcrowding in this hospital. The beds themselves would be regarded by our nurses with great disfavour, for they all have side-pieces rising well up to the top level of the bed-clothes, which are thus enclosed on all sides by iron railings. It cannot be easy to make beds or nurse patients in such boxes. This hospital is much frequented by medical students, and so far as their requirements are concerned the institution is well equipped. Excellent laboratories and lecture theatres are available, and the most is made of the limited accommodation. In the operating theatre it was very noticeable that chemical disinfectants were freely employed, though among the operating surgeons there was much divergence of method, more even than among London surgeons so far as theatre technique is concerned.

### The New Rijks Hospitalet, Copenhagen.

In Copenhagen, on the other hand, the new Rijks Hospitalet may justly be compared with the Rudolf Virchow Krankenhaus. It is, indeed, built on a similar plan; a number of isolated buildings are situated in pleasant grounds of large extent. Interiorly the wards are splendidly planned, with a fine disregard for economising space. Many of them have beds in fours, placed in large three-sided spaces opening into a wide passage, on the other side of which other groups of four beds are similarly placed, but not exactly opposite the group on the first side. This arrangement calls for a building of considerable width and allows plenty of fresh air for all the patients. The furnishing of the wards is carried out on a fairly luxurious scale, and the washing basins and fittings are a delight to use. An adjoining day room is provided with a surprisingly good quality of tables and chairs; in fact equal to that to be found in a good club in this country. In Denmark one could not fail to be impressed by the general prevalence of the German model in hospital matters, although, of course, in everyday life there is no love lost between the Danes and their Teutonic neighbours. Naturally this new hospital is exceptionally well favoured; among the several other institutions for the sick in Copenhagen there are some which can hardly be described as up to date. To cite an example, there is the Fever Hospital, the Blegdams Hospitalet. Here the wards are on a very much smaller scale—much too cramped for fever cases. There is, however, a considerable degree of subdivision and isolation, and the patients are accommodated in numerous small pavilions. Those for convalescents are distinctly inconvenient and lacking in space. It was not a little surprising to see a considerable number of temporary buildings—some quite newly erected. They reminded one forcibly of cricket-club pavilions in this country, and must prove expensive to keep in good repair and difficult to warm in winter time. Such erections are in this country recognised to be disadvantageous in many ways and only resorted to by improvident local authorities or in times of the greatest urgency.

Copenhagen possesses a far larger accommodation for venereal diseases in proportion to the size of the town than does any town in this country, and the chief institution used as a lock hospital is the Rudolf Bergs Hospitalet. This institution is not modern but represents a good average. Here the beds are somewhat closely packed, but nursing and administration are distinctly efficient.

